# Whole-Genome Sequencing and Drug-Susceptibility Analysis of Serial *Mycobacterium abscessus* Isolates from Thai Patients

**DOI:** 10.3390/biology11091319

**Published:** 2022-09-05

**Authors:** Orawee Kaewprasert, Ditthawat Nonghanphithak, Ploenchan Chetchotisakd, Wises Namwat, Rick Twee-Hee Ong, Kiatichai Faksri

**Affiliations:** 1 Department of Microbiology, Faculty of Medicine, Khon Kaen University, Khon Kaen 40002, Thailand; 2 Research and Diagnostic Center for Emerging Infectious Diseases (RCEID), Khon Kaen University, Khon Kaen 40002, Thailand; 3 Department of Medicine, Srinagarind Hospital, Faculty of Medicine, Khon Kaen University, Khon Kaen 40002, Thailand; 4 Saw Swee Hock School of Public Health, National University of Singapore and National University Health System, Singapore 117549, Singapore

**Keywords:** mutation rate, *Mycobacterium abscessus*, phylogenetic analysis, whole genome sequence, drug resistance

## Abstract

**Simple Summary:**

We undertook genetic diversity analysis between subspecies, mutation-rate estimation and identification of drug-resistant mutations analysis of *Mycobacterium abscessus*. We differentiated between re-infection and persistent infection of *M. abscessus* based on genetic distance using WGS analysis. We found a high mutation rate of *M. abscessus* during the initial infection process, followed by a dramatic reduction in this rate in serial isolates separated by more than 180 days. Mutation rate also differed between the two subspecies that we included: subsp. *abscessus* (MAB) and subsp. *massilense* (MMAS). MMAS strains show more susceptibility to clarithromycin, amikacin and linezolid than MAB. This can inform choice of an appropriate regimen for patient management and reduce the speed of development of drug resistance for *M. abscessus* patients.

**Abstract:**

*Mycobacterium abscessus* is an important pathogen that can cause serious human diseases and is difficult to treat due to antibiotic resistance. In this study, we analyzed, using whole-genome sequence (WGS) data, *M. abscessus* strains serially isolated from patients at various time intervals. We undertook genetic diversity analysis between subspecies, mutation-rate estimation and identification of drug-resistant mutations with minimum inhibitory concentration (MIC) analysis. Clonal isolates of *M. abscessus*:—subsp. *abscessus* (MAB) and subsp. *massiliense* (MMAS)—causing persistent infection through time, differed by 0–7 and 0–14 SNPs, respectively, despite being isolated 1 to 659 days apart. Two cases caused by MMAS differed by ≥102 SNPs at 350 days apart and were regarded as examples of reinfection. Isolates collected ≤7 days apart exhibited a high mutation rate (133.83 ± 0.00 SNPs/genome (5 Mb)/year for MMAS and 127.75 SNPs/genome (5 Mb)/year for MAB). Mutation rates declined in a time-dependent manner in both subspecies. Based on isolates collected > 180 days apart, MMAS had a significantly higher average mutation rate than MAB (2.89 ± 1.02 versus 0.82 ± 0.83 SNPs/genome (5 Mb)/year, (*p* = 0.01), respectively). All well-known drug-resistance mutations were found to be strongly associated with high MIC levels for clarithromycin and ciprofloxacin. No known mutations were identified for strains resistant to linezolid and amikacin. MAB strains in the study were susceptible to amikacin, while most MMAS strains were susceptible to clarithromycin, amikacin and linezolid. No hetero-resistance was found in the strains analyzed. Our study reports the genetic diversity and mutation rate of *M. abscessus* between the two major subspecies and confirms the drug resistance-associated mutations. Information about drug-resistance and associated mutations can be applied in diagnosis and patient management.

## 1. Introduction

*Mycobacterium abscessus* is a rapidly growing species of nontuberculous mycobacteria, which is classified into three subspecies: *M. abscessus* subsp. *abscessus* (MAB); *M. abscessus* subsp. *massiliense* (MMAS); and *M. abscessus* subsp. *bolletii* (MBOLL) [1]. *Mycobacterium abscessus* is an emerging pathogen that causes a wide spectrum of diseases including respiratory infection, disseminated disease, soft-tissue and bone infection. It is also one of the most multidrug-resistant mycobacteria with cure rates often below 50% despite a combination therapy of clarithromycin and amikacin for 6–12 months or more [2,3].

Infections with *M. abscessus* can re-occur within the same individual, a process caused by two mechanisms. The first is relapse or persistent infection (PI) with the same clonal strain infecting the same patient through time [4]. The second is reinfection (RI) by an exogenous strain [4]. These two forms of infection differ in their implications for patient management and control. In cases of persistent infection there is a need to adjust the treatment regimen, whereas in reinfection cases there is a need to control the source of infection [5]. When treatment of *M. abscessus* appears to have failed, it is important for epidemiological and patient-management strategies to be able to distinguish between RI and PI. This is best done by studying serially isolated strains from the patient. Genome comparisons of the serially isolated bacteria can be used to differentiate between RI and PI. There have only been a few such studies on *M. abscessus* using whole-genome sequencing (WGS) analysis and the strains examined were all found to be instances of PI [6,7]. However, it is still unclear what the threshold should be for determining genetic relatedness to distinguish between PI and RI by the number of SNP differences [6,8,9]. Most of the isolates of *M. abscessus* that have been examined for genetic diversity are from cystic fibrosis (CF) patients, a clinical scenario that might differ from that in Thailand.

Mutations are genetic changes necessary for evolution to proceed and the mutation rate is the frequency of new mutations in a single gene or organism over time [10,11]. Evolution of bacteria relies on the constant fine-tuning of their mutation rates, which optimizes their adaptability to changing environmental conditions [12]. Although high genetic diversity within subspecies of *M. abscessus* has been suggested [9,13], the mutation rates in different subspecies has never been clearly reported. 

During persistent infection, hetero-resistance can occur if the infecting strain has two populations, one drug susceptible while the other is drug resistant carrying specific resistance-associated mutations. Such populations can confound clinical susceptibility testing and subsequent antibiotic treatment [14,15]. One previous study has identified heterogeneous patterns using GenoType NTM-DR assays [16] in such mixed *M. abscessus* populations. However, no study has examined hetero-resistance based on variant frequencies of known drug-resistance mutations together with minimum inhibitory concentration (MIC) levels. Identification of hetero-resistance, and of the mutations involved, is important to avoid life-long treatment with inappropriate antibiotics and the concomitant risk of further development of resistance.

In this study, we therefore aimed to determine genetic diversity and mutation rates, using WGS analysis, of *M. abscessus* serially isolated from the same patients with known PI and RI due to MAB and MMAS subspecies, from Thailand. We also aimed to investigate hetero-resistance based on variant frequencies in subpopulations and association between mutations and MIC values.

## 2. Materials and Methods

### 2.1. Clinical Mycobacterium abscessus Isolates

#### 2.1.1. *Mycobacterium abscessus* Collection and Study Setting 

Sixty-nine clinical *M. abscessus* isolates from 26 patients in the period 2012–2017 were studied. Isolates had been maintained as stock cultures at the Clinical Microbiology Laboratory in Srinagarind Hospital, Khon Kaen University, Thailand. Cases of true infection and of colonization were differentiated using published guidelines [4]. Distinguishing between RI and PI, as well as subspecies identification, was based on MLST, as described previously [4]. This study was approved by Khon Kaen University Ethics Committee for Human Research (EC. No. HE591454).

#### 2.1.2. Bacterial Culture and DNA Extraction

*Mycobacterium abscessus* isolates were inoculated on Löwenstein-Jensen (LJ) media and incubated at 37 °C for 7 days. The cetyl-trimethyl-ammonium bromide-sodium chloride (CTAB) [17] method was used for genomic DNA extraction. This involves initial incubation of cultured *M. abscessus* colonies with 50 mg/mL lysozyme, followed by 10% SDS, 10 mg/mL proteinase K and CTAB to disrupt the cell membranes. Cold absolute ethanol and 5M NaCl were added to precipitate DNA. DNA samples were re-suspended in TE buffer and stored at −20 °C until use.

### 2.2. Identifying Recurrent Infection of M. abscessus

#### 2.2.1. Whole-Genome Sequencing

All DNA samples were subjected to Illumina high-throughput sequencing at NovogeneAIT Genomics, Singapore. A total amount of 1 μg DNA per sample was used as input material for the DNA sample preparations. The library preparations were sequenced on an Illumina HiSeq (San Diego, CA, USA) platform and paired-end reads of 2 × 150 bp were generated. 

#### 2.2.2. Analysis Pipeline for WGS

The quality of raw sequences was checked using FastQC version 0.11.7 [18]. Trimmomatic (v0.36) software [19] was used to remove low-quality reads (leading:3, trailing:3, sliding window:4:15 and minlen:75). High-quality paired-end reads were then mapped to the M. abscessus ATCC 19977 reference genome (GenBank accession number CU458896.1) using BWA-mem (v.0.7.17) [20]. For converting SAM to BAM format, sorting and indexing the bam files, SAMtools v0.1.19 algorithm was used [21]. GATK version 4.0.5.2 [22] was used for realignment, generating coverage statistics and mapping details. Both GATK and SAMtools were used for variant calling and filtering, including identification of single-nucleotide polymorphisms (SNPs) and small indels. Variants were filtered by having minimum base quality (Q):Q ≥ 30, minimum mapping quality score (C):C ≥ 40, SNP quality (QSNP):QSNP ≥ 30, depth of coverage (d):d ≥ 60 and frequencies of reads supporting SNPs ≥ 80%. 

#### 2.2.3. Phylogenetic Analysis 

These highly stringent parameters (Q30, C40, QSNP30, 60X and ≥80% frequency of the main variants) were used to generate high-confidence SNPs. The WGS-based phylogeny was based on an alignment of 1230 high-confidence SNPs among strains, generated from mpileup, VCF (based on the common variant set from GATK and SAMtools) and coverage files. Maximum-likelihood (ML) analysis was performed using MEGA-7 [23] with the general time-reversible (GTR) and gamma model. Support for individual nodes was assessed using 1000 bootstrap replicates. The phylogenetic tree was visualized using *iTol software* [24]. Subspecies (MAB and MMAS) were identified based on the *erm*(41) gene sequences, phylogenetic tree and MLST data obtained from a previous study [4,25].

### 2.3. Mutation Rate Analysis 

#### 2.3.1. Mutation Rate Estimation 

The mutation rate was determined based on the number of SNPs (N) that differed between sequential isolates from individual patients, scaled by genome size (5 Mb) and time between collection of isolates (years). Following [26], the mutation rate = $\frac{N}{\left[\left(\mathrm{genome}\;\mathrm{size}\right)\times \left(\mathrm{days}\;\mathrm{of}\;\mathrm{interval}\;\mathrm{time}/365\right)\right]}$.

#### 2.3.2. Statistical Comparisons of Mutation Rates

Comparisons between mutation rates in MAB and MMAS were done using independent *t*-tests in SPSS version 19.0 (New York, NY, USA). A value of *p* < 0.05 indicates a statistically significant result.

### 2.4. Drug-Resistance Analysis

#### 2.4.1. Drug-Susceptibility Testing Using the Broth-Microdilution Method

Three to five colonies were selected on the LJ media using a loop and emulsified in demineralized water by vortexing with glass beads until a homogeneous suspension was obtained. If large clumps remained, only the supernatant was used. The suspension was adjusted to 0.5 McFarland standard. Fifty microliters of the suspension was transferred into a tube of cation-adjusted Mueller–Hinton broth with TES buffer to 5 × 10^5^ cfu/mL concentration and mixed well. Then, 100 µL of this mixture was added to each well of a RAPMYCOL Sensitire 96-well plate (Sensitire, Trek Diagnostic Systems, United Kingdom) and the plate was covered with an adhesive seal. MIC plates were incubated at 37 °C in non-CO_2_ conditions and examined on days 3, 5 and 14.

#### 2.4.2. Known Mutations Related to Drug Resistance 

WGS data were searched for mutations associated with drug resistance (Supplementary Table S4) by comparing the extracted base calls from the vcf files to known resistance-conferring alleles using in-house python scripts. 

## 3. Results

### 3.1. Study Population and Study Setting 

Sixty-nine clinical *M. abscessus* isolates were serially isolated from 26 patients referred to Srinagarind Hospital, Thailand, during 2012–2017. These patients had an average age of 54.39 ± 15.41 years and five were male (19.23%). Based on clinical criteria described previously [4], 22 of the 26 cases (MAB *n* = 9 and MMAS *n* = 13) were true infections and four cases were due to colonization (MAB *n* = 3 and MMAS *n* = 1).

### 3.2. Distinguishing between Persistent and Re-infection Cases in Recurrent M. abscessus Infection and Cluster Analysis

**Figure 1:**
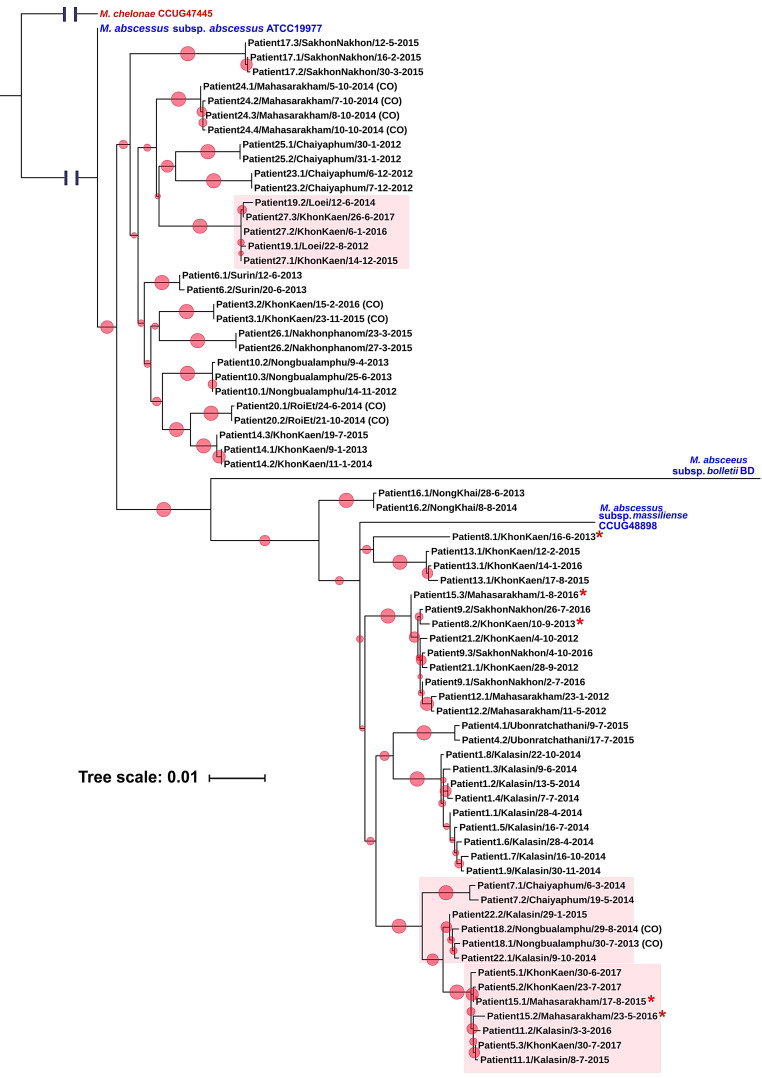
Phylogenetic tree of 69 isolates of *M. abscessus* causing persistent infection or re-infection based on 1230 high-confidence SNPs from WGS analysis. Relationships between serial isolates as depicted in this tree were the basis for distinguishing between persistent infection (PI) and re-infection (RI). The phylogeny was inferred using the maximum likelihood method with 1000 bootstrap replicates. Each red circle represents a bootstrap value and the size of the circle is proportional to its value (the largest circles represent 100%). Reference strains of each *M. abscessus* subspecies were included. *Mycobacterium chelonae* CCUG47445 was used as the outgroup. Red boxes enclose four clusters of possible transmission events. Red asterisks indicate the re-infection of Patient #8 and Patient #15. “CO” in brackets refers to instances of colonization.

The generated phylogenetic tree identified clusters representing clonal lineages of isolates (Figure 1). Serial isolates differing at few SNP sites, were regarded as examples of PI. The cut-off value for identifying PI differed between the two subspecies, MAB (0–7 SNPs) and MMAS (0–14 SNPs). These values were based on biological ground-truthing criteria: one-day interval between paired isolates from the same patient, known subspecies (MLST, truncation of *erm*(41) gene in MMAS and results of clarithromycin susceptibility tests. In two cases (Patient #8 and Patient #15) infected by MMAS, their serial isolates differed at many SNPs (≥102 SNPs) and were far apart on the SNP phylogenetic tree, indicating likely replacement of one clone by another, and hence RI (Table 1).

### 3.3. Estimation of Mutation Rate of M. abscessus during Persistent Infection (PI) and Persistent Colonization 

**Figure 2:**
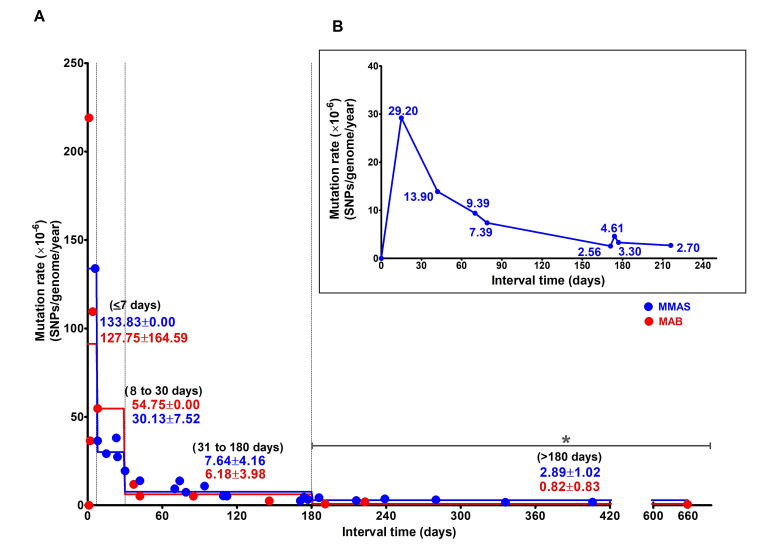
Estimated mutation rates of *M. abscessus* during persistent infection and persistent colonization. (**A**) Estimated average *M. abscessus* mutation rates obtained by comparing numbers of SNPs among strains causing persistent infection and serially isolated at various intervals (25 patients, *n* = 66 isolates). Data points represent four intervals; ≤7 days, 8–30 days, 31–180 days and >180 days to determine *M. abscessus* mutation rate in two subspecies: MAB (red) and MMAS (blue). The mutation rate of MMAS was significantly higher than MAB at isolation intervals exceeding 180 days. Mutation rates are shown as mean ± SD, * *p* = 0.01 (independent *t*-test). (**B**) Average MMAS mutation rate from a single patient with 9 serial isolates (with isolation times of 15 days, 42 days, 70 days, 79 days, 171 days, 174 days, 177 days and 216 days after initial infection). A decline in numbers of SNPs with increasing time intervals was found, similar to the multiple-isolate analysis in (**A**).

Mutation rate was determined from numbers of SNPs that had changed between sequential isolates causing PI (*n* = 66/69 isolates, from 25 patients). Isolates collected ≤7 days apart exhibited a high mutation rate (133.83 SNPs/genome (5 Mb)/year for MMAS and 127.75 ± 164.59 SNPs/genome (5 Mb)/year for MAB). Paired strains separated by 8–30 days, 31–180 days and >180 days exhibited lower mutation rates in both subspecies (Figure 2A and Supplementary Table S2). MMAS had a significantly higher average mutation rate based on isolates collected >180 days apart than did MAB (2.89 ± 1.02 and 0.82 ± 0.83 SNPs/genome (5 Mb)/year, (*p* = 0.01), respectively.

The nine serial isolates of MMAS from Patient #1 confirmed the dramatic decrease in number of SNP sites changing from the initial seven days post-infection (29.20 SNPs/ genome (5 Mb)/year) to a low mutation rate (2.70 SNPs/genome (5 Mb)/year) after 180 days (Figure 2B and Supplementary Table S2). 

### 3.4. Drug Resistance-Conferring Mutations Related to MIC Valuese

#### 3.4.1. Clarithromycin

**Figure 3:**
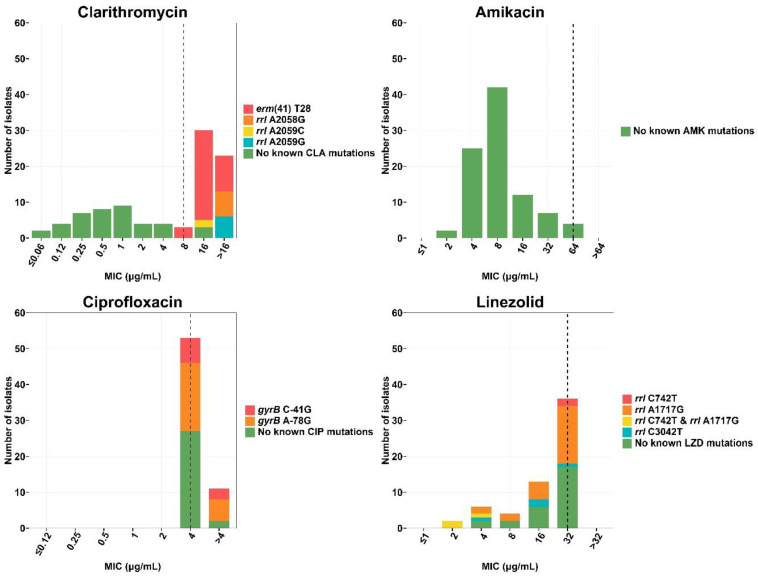
Distributions of drug resistance-conferring mutations with MIC values. Histogram represents the collection of isolates colored by different variants on the y-axis. The dashed lines indicate MIC breakpoints that are considered to indicate resistance.

**Figure 4:**
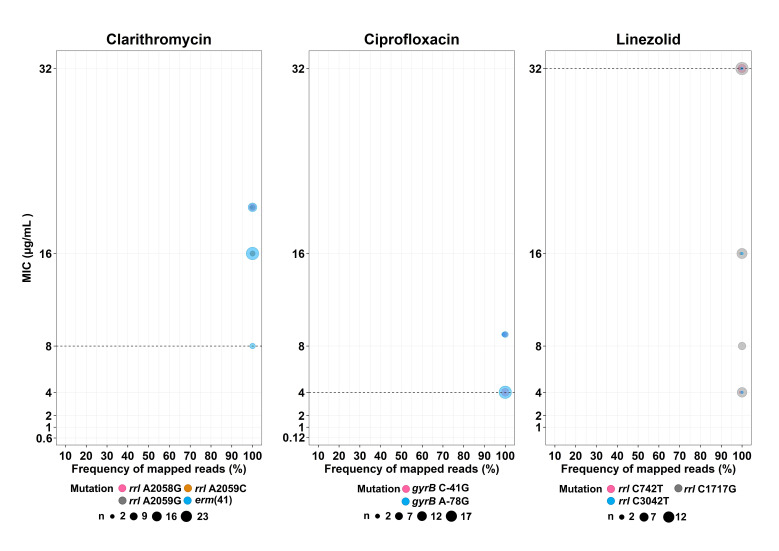
Comparisons between hetero-resistance (inferred from read frequencies of relevant SNPs) and MIC levels. The dashed lines indicate MIC breakpoints that are considered to indicate resistance. Each circle is proportional to the number of isolates.

The *erm*(41) gene corresponding to natural clarithromycin resistance was found only in MAB (*n* = 32/69 isolates, 46.38%) and is associated with high MIC values; 8 µg/mL (*n* = 3), 16 µg/mL (*n* = 19) and >16 µg/mL (*n* = 10). Acquired clarithromycin-resistant strains having mutations in the *rrl* gene were mostly found in MMAS (*n* = 9). Well known *rrl* mutations, A2058G, A2059C and A2059G (*Escherichia coli* numbering system), were detected in isolates with high MIC values ≥16 µg/mL. All but four of the phenotypically resistant isolates were found to harbor known resistance-associated mutations, while these were absent from all the susceptible isolates (Figure 3, Supplementary Tables S3 and S4). In addition, all clarithromycin-resistant isolates had 99–100% of the sequence reads supporting the presence of the resistance allele. Thus, no hetero-resistance for clarithromycin was found in any isolate (Figure 4).

#### 3.4.2. Amikacin

Most of the strains were phenotypically susceptible to amikacin (*n* = 65, 94.20%). while the four isolates with high MIC values for amikacin were not found to harbor any known resistance-conferring mutations in the *rrs* gene (Figure 3, Supplementary Tables S3 and S4).

#### 3.4.3. Ciprofloxacin

All the 69 isolates in this study were phenotypically resistant to ciprofloxacin, with 55.07% (*n* = 38) harboring well-known resistance mutations in the QRDR regions within *gyrA* and *gyrB* (Supplementary Table S4), having the C-41G and A-78G sequevars in 12 and 26 isolates, respectively. No hetero-resistance was found at these two mutations (Figure 4).

#### 3.4.4. Linezolid

Of the 69 *M. abscessus* isolates, 7.25% (*n* = 5) were MMAS harboring C742T (*M. abscessus* numbering), which was found only in the resistant-phenotype group with an MIC value of 32 µg/mL (Figure 3 and Supplementary Table S3). However, other mutations, including A1717G and C3042T, were identified in both susceptible and resistant strains. No hetero-resistance for this particular drug was detected: 98.7–100% of reads supported the same alleles (Figure 4). No significant resistance-associated mutations from 23s rRNA genes and related genes including *rplC*, *rplD* and *rplV* were detected in linezolid-resistant strains. Note that these mutations can be detected in both susceptible and resistant phenotypes.

## 4. Discussion

Human infection with *M. abscessus* is most often due to environmental exposure [1]. It has thus been questioned whether human-to-human transmission of this pathogen can occur [27]. While transmission of *M. abscessus* between humans cannot be excluded, we did not find clear evidence to support this phenomenon in our study. We observed three clusters of possible transmission events in our study population. The largest cluster comprised five patients who lived in three provinces in the same region (Khon Kaen, Mahasarakham and Sakhon Nakhon) during 2015–2017. Four of these patients had pulmonary infection, consistent with transmission, but one patient had a bone-marrow infection. Based on our hospital information system, the five patients had no familial links. The only epidemiological link among these patients was the receipt of medical care in the same hospital. As *M. abscessus* is an environmental pathogen, these individuals could have been infected from the same source, such as from the same hospital environment, which would similarly display such a pattern. 

We found that the cut-off values for identifying persistent infections of MAB and MMAS are 0–7 and 0–14 SNPs, respectively. These values are slightly lower than the previously reported <20–25 SNPs between strains in a transmission analysis [8,9]. These differences might be due to the different analysis parameters used and/or the setting of the study (serially isolated strains from single patients versus suspected transmitted cases). We also found SNP cut-off differences between subspecies. Isolates derived from persistent infections with MMAS differed in higher numbers of SNPs than was the case for MAB. Recombination occurs more frequently in MMAS than MAB [28,29], which might explain such differences between the two subspecies. Recombination could also contribute virulence and resistance genes that could allow MMAS to better adapt inside the host body during persistent infection [30].

A previous study has reported a mean mutation rate using Bayesian analysis for MAB and MMAS of 1.8 and 0.47 SNPs per genome per year, respectively [8]. In our study, we used the time intervals between serial isolates to estimate mutation rates and also found that these differed between the two subspecies (2.9 and 0.8 SNPs per genome per year for MAB and MMAS, respectively, after 180 days). Initially, we incorrectly assumed that very few spontaneous mutations would occur during the short time (<7 days) of the initial infection and adaptation process. In 24/26 of our cases, patient medical records indicated that the first isolate collected was early in the infection process (one case had earlier rapid-growing mycobacterial infection and no data were available for another). However, a preexisting colonization before the onset of disease cannot be excluded. Over longer time spans, we unexpectedly found that both MAB and MMAS exhibited a decline in the number of mutations. Reduced mutability controlled by the mutator gene (*mutT*) has been reported after long-term in vitro cultivation of *E. coli* [31]. A steady increase in mutations might be harmful to the bacterium due to an increase in deleterious mutations [32], resulting in selection for a lower mutation rate [33,34]. At the beginning of the infection process (such as <7 days), mixed-strain infection might lead to a higher number of SNPs being detected. The subsequent apparent decline of the mutation rate might be due to the rise of a dominant clone. However, we observed a similar time-dependent decline in mutation rate among all PI cases. We found no evidence of mixed infection in our study, nor have any been reported previously. Possibly, the decreasing apparent mutation rate we observed might be due to selection of subpopulations of *M. abscesses* from serial isolates that differed in their ability to grow in culture media. The observed decline of mutation rate might occur when the pathogen has entered into an equilibrium state. Such a time-dependency of mutation rate was found in both subspecies but was more pronounced in MMAS after 180 days. Another possible explanation for the decline in mutation rate might be a difference between environmental and within-host conditions. The difference between the two subspecies might explain the reported lower response to drug treatment by MAB [1,35,36]. We confirmed the dramatic decline in mutation rates using the nine clinical samples collected from Patient #1 over a period of 216 days. Although subspecies *bolletii*, which is rare in Thailand, was not included in our analysis, it shares some genetic similarities with subspecies *massiliense* [37] and the mutation rate might be similar. 

We analyzed the drug resistance-conferring mutations in the context of MIC values. All clarithromycin-resistant strains had the *erm*(41) T28 mutation and most isolates had MIC values of 16 µg/mL. Previous studies have reported that all *erm*(41) T28 sequevars are resistant to clarithromycin with MIC values ranging from 8 to ≥16 µg/mL [38,39,40]. This mutation is a synonymous substitution associated with slightly lower MIC values in some isolates [41]. We also found that *rrl* mutations (A2058G, A2059C and A2059G) were associated with clarithromycin resistance with higher MICs (>16 µg/mL). These mutations in the ribosomal RNA decrease affinity of the drug to bind to at domain V of the 23S rRNA [42], which in turn, is reflected in higher bacterial MIC values.

For amikacin, we found that 4 of 69 isolates had high MIC values (64 µg/mL) but harbored no known amikacin-resistant mutations. The discordance between phenotypic and genotypic susceptibilities are likely due to these patients being treated with aminoglycosides or due to unidentified resistance-associated mutations in other genes [43].

Ciprofloxacin is an antibiotic belonging to the fluoroquinolone class, where resistance has been found mainly related to the QRDR region in the *gyrA* and *gyrB* genes, and mutations within the promoter region upstream of the beginning of *gyrB*; C-41G and A-78G [43]. Despite a previous study suggesting that these two mutations have only low-confidence association with resistance [43], we found that they were present in 55.07% (*n* = 38) of ciprofloxacin-resistant isolates. As we did not find any other resistance- associated mutation in these isolates, our study supported that *gyrB* C-41G and A-78G are associated with ciprofloxacin resistance. We found no ciprofloxacin-resistant isolates harboring *gyrB* Ala90Val and Asp96Asn mutations. A previous study from southern Brazil [44] reported that Ala90Val was present in 88.5% (31 of 35) ciprofloxacin-resistant MMAS strains there. Presumably strains of MMAS differ between the two regions.

Linezolid is reportedly one of the most potent antibiotics against infections caused by *M. abscessus* [45]. Based on our analysis of 23S rRNA gene and ribosomal proteins (L3, L4, and L22), we did not find previously reported resistance-associated mutations, suggesting that relevant mutation(s) occurred outside the 23S rRNA region and associated genes.

From our drug-resistance analysis above, most of the isolates we studied presented high MIC values, conferring resistance to antibiotics of choice for *M. abscessus* treatment. This pathogen is well-known as difficult to treat [46,47]. Therefore, advanced computational tools, such as machine learning, have been applied for drug discovery to investigate novel compounds such as 1-(phenylsulfonyl)-1H-benzimidazol-2-amines [48]. Another novel target is phosphopantetheine adenylyltransferase, identified using fragment-based drug design [49]. Additional candidate drugs have been reported, such as compounds that potentially interfere with mycobacterial membrane protein large 3 (MmpL3) which transports mycolic acid in mycobacteria [50]. Drug-discovery studies, including those mentioned above, can be expected to lead to improved treatment for *M. abscessus* in the future.

We also analyzed the association between genotypic hetero-resistance, based on the proportion of WGS mapped reads of resistance-conferring SNPs, and MIC levels. This kind of analysis might be important for early detection of slight MIC changes in the bacterial population during persistent infection because of long-term treatment. Such changes might be a consequence of the hetero-resistance phenomenon, although we found no evidence of this in *M. abscessus*.

Our study had some limitations. Despite collecting specimens for six years (2012–2017) our sample size was relatively small, and the isolates were collected from different body sites which might have contributed to their diversity. In most cases, we obtained 2–4 samples per patient. The collection period of the samples in our study was not updated which might affect some results such as the distribution of drug-resistant strains and MIC. Longitudinally collected samples with multiple time points and increased sample size would provide a better estimate of mutation-rate changes through time and more opportunity to investigate and understand hetero-resistance. To verify the phenomenon of declining mutation rate through time, it is necessary to ensure that mixed strains, including mixed subpopulations from the primary infection, are not present in our samples. Single-cell sequencing is required to gain further insight into the hetero-resistance phenomenon and ensure the absence of a mixture of subpopulations. Due to the limited budget, the verification of mixed subpopulations from the primary infection was not included in our study. To discover additional novel mutations associated with antibiotic resistance in *M. abscessus*, pan-susceptible strains will need to be compared with strains resistant to specific antibiotics.

## 5. Conclusions

In conclusion, we reported the cut-off values for numbers of SNPs to differentiate between PI and RI, which differed between MAB and MMAS, two subspecies of *M. abscessus*. High mutation rates occurred for both MAB and MMAS during the initial infection process, but the rates decreased after 180 days post-infection and were quantitatively different between subspecies. These differences might explain their differing adaptability and response to treatment. We characterized the distribution of resistance-related genes and MIC values of drugs of choice for treatment of *M. abscessus.* Most MAB isolates were susceptible to amikacin and most MMAS strains were susceptible to clarithromycin, amikacin and linezolid. Appropriate choice of drug for treatment is an important step in delaying the evolution of drug resistance. High MIC levels for clarithromycin and ciprofloxacin were associated with known mutations, while no mutations conferring linezolid and amikacin resistance were found. More mutation targets are required to increase the sensitivity for detection of drug resistance, especially for drugs of choice for *M. abscessus* treatment.

## Figures and Tables

**Table 1:** SNP cut-off values used to differentiate between re-infection and persistent infection caused by *M. abscessus*.

Infection Status	*M. abscessus* ^a^	
	MAB	MMAS
Persistent infection (PI)	≤7 SNPs (*n* = 9)	≤14 SNPs (*n* = 11)
Re-infection (RI)	NA	≥102 SNPs (*n* = 2)

^a^ MAB = *M. abscessus* subsp. *abscessus*; MMAS = *M. abscessus* subsp. *massiliense*; and NA = Not available.

## Data Availability

The datasets generated and/or analyzed during the current study are available in the NCBI repository, containing 69 biosamples under the bioproject accession No. PRJNA523980.

## Supplementary Materials

The following are available online at https://www.mdpi.com/article/10.3390/biology11091319/s1, Table S1: Mutation rate of serial isolates of *M. abscessus*, Table S2: Estimation of *M. abscessus* mutation rates at different time intervals relative to the initial isolate, Table S3: Frequency and MIC-distribution of isolates with drug resistance-conferring mutations, Table S4: Literature of mutation associated *M. abscessus* drug resistance.
