# Supplementary material for: Whole-Genome Sequencing and Drug-Susceptibility Analysis of Serial Mycobacterium abscessus Isolates from Thai Patients
Source: Biology (Basel). 2022 Sep 5;11(9):1319. doi: 10.3390/biology11091319 (PMC9495349; doi:10.3390/biology11091319)
Supplement: Supplementary file 1 [file biology-11-01319-s001.zip › biology-1877284-supplementary.pdf]

Supplementary Table S1: Mutation rate, calculated as stated in the text, of serial isolates of *Mycobacterium abscessus*.

Patient number	Subspecies	Dates of isolation (dd/mm/yy)	Interval time (from the initial isolate)	SNP differences between successive pairs of isolates	Mutation rate calculated since the initial isolate SNP/genome size (5Mb)/year ($x10^{-6}$)	Type of infection
P1.1	*massiliense*	28/4/14	-			Persistent
P1.2	*massiliense*	13/5/14	15	6	29.200	Persistent
P1.3	*massiliense*	9/6/14	42	8	13.905	Persistent
P1.4	*massiliense*	7/7/14	70	9	9.386	Persistent
P1.5	*massiliense*	16/7/14	79	8	7.392	Persistent
P1.6	*massiliense*	16/10/14	171	6	2.561	Persistent
P1.7	*massiliense*	19/10/14	174	11	4.615	Persistent
P1.8	*massiliense*	22/10/14	177	8	3.299	Persistent
P1.9	*massiliense*	30/11/14	216	8	2.704	Persistent
P3.1	*abscessus*	23/11/15	-			Colonization
P3.2	*abscessus*	15/2/16	84	1	0.869	Colonization
P4.1	*massiliense*	9/7/15	-			Persistent
P4.2	*massiliense*	17/7/15	8	4	36.500	Persistent
P6.1	*abscessus*	12/6/13	-			Persistent
P6.2	*abscessus*	20/6/13	8	6	54.750	Persistent
P7.1	*massiliense*	6/3/14	-			Persistent
P7.2	*massiliense*	19/5/14	74	14	13.811	Persistent
P8.1	*massiliense*	18/6/13	-			Re-infection
P8.2	*massiliense*	10/9/13	84	140	121.667	Re-infection
P9.1	*massiliense*	2/7/16	-			Persistent
P9.2	*massiliense*	26/7/16	24	9	27.375	Persistent
P9.3	*massiliense*	4/10/16	94	14	10.872	Persistent

Supplementary Table S1: Mutation rate, calculated as stated in the text, of serial isolates of *Mycobacterium abscessus. (Cont.)*

Patient number	Subspecies	Dates of isolation (dd/mm/yy)	Interval time (from the initial isolate)	SNP differences between successive pairs of isolates	Mutation rate calculated since the initial isolate SNP/genome size (5Mb)/year ($x10^{-6}$)	Type of infection
P10.1	*abscessus*	14/11/12	-			Persistent
P10.2	*abscessus*	9/4/13	146	5	2.500	Persistent
P10.3	*abscessus*	25/6/13	223	6	1.964	Persistent
P11.1	*massiliense*	8/7/15	-			Persistent
P11.2	*massiliense*	3/3/16	239	12	3.665	Persistent
P12.1	*massiliense*	23/1/12	-			Persistent
P12.2	*massiliense*	11/5/12	109	8	5.358	Persistent
P13.1	*massiliense*	12/2/15	-			Persistent
P13.2	*massiliense*	17/8/15	186	11	4.317	Persistent
P13.3	*massiliense*	14/1/16	336	8	1.738	Persistent
P14.1	*abscessus*	9/1/13	-			Persistent
P14.2	*abscessus*	2013/1/11	2	1	36.500	Persistent
P14.3	*abscessus*	19/7/13	191	2	0.764	Persistent
P15.1	*massiliense*	17/8/15	-			Persistent
P15.2	*massiliense*	23/5/16	280	12	3.129	Persistent
P15.3	*massiliense*	1/8/16	350	102	21.274	Re-infection
P16.1	*massiliense*	28/6/13	-			Persistent
P16.2	*massiliense*	8/8/14	406	10	1.798	Persistent
P17.1	*abscessus*	16/2/15	-			Persistent
P17.2	*abscessus*	30/3/15	42	3	5.214	Persistent
P17.3	*abscessus*	12/5/15	85	6	5.153	Persistent
P18.1	*massiliense*	30/7/13	-			Colonization
P18.2	*massiliense*	29/8/14	395	5	0.924	Colonization
P19.1	*abscessus*	22/8/12	-			Persistent
P19.2	*abscessus*	12/6/14	659	5	0.554	Persistent

Supplementary Table S1: Mutation rate, calculated as stated in the text, of serial isolates of *Mycobacterium abscessus.* (Cont.)

Patient number	Subspecies	Dates of isolation (dd/mm/yy)	Interval time (from the initial isolate)	SNP differences between successive pairs of isolates	Mutation rate calculated since the initial isolate SNP/genome size (5Mb)/year ($x10^{-6}$)	Type of infection
P20.1	*abscessus*	24/6/14	-			Colonization
P20.2	*abscessus*	21/10/14	119	5	3.067	Colonization
P21.1	*massiliense*	28/9/12	-			Persistent
P21.2	*massiliense*	4/10/12	6	11	133.833	Persistent
P22.1	*massiliense*	9/10/14	-			Persistent
P22.2	*massiliense*	29/1/15	112	8	5.214	Persistent
P23.1	*abscessus*	6/12/12	-			Persistent
P23.2	*abscessus*	7/12/12	1	0	0.000	Persistent
P24.1	*abscessus*	5/10/14	-			Colonization
P24.2	*abscessus*	7/10/14	2	4	146.000	Colonization
P24.3	*abscessus*	8/10/14	3	2	48.667	Colonization
P24.4	*abscessus*	10/10/14	5	5	73.000	Colonization
P25.1	*abscessus*	30/1/12	-			Persistent
P25.2	*abscessus*	31/1/12	1	3	219.000	Persistent
P26.1	*abscessus*	23/3/15	-			Persistent
P26.2	*abscessus*	27/3/15	4	6	109.500	Persistent
P27.1	*abscessus*	25/4/16	-			Persistent
P27.2	*abscessus*	1/6/16	37	6	11.838	Persistent
P27.3	*abscessus*	26/6/17	427	0	0.000	Persistent
P28.1	*massiliense*	30/6/17	-			Persistent
P28.2	*massiliense*	23/7/17	23	12	38.087	Persistent
P28.3	*massiliense*	30/7/17	30	8	19.467	Persistent

Supplementary Table S2: Estimation of *Mycobacterium abscessus* mutation rates at different time intervals relative to the initial isolate.

Subsp.	No. of SNPs	Interval time (days)	Mutation rate ($\times 10^{-6}$)	Mutation rate ($\times 10^{-6}$), Mean (SD)
**≤ 7 days**				
abscessus	1	2	36.50	127.75 (164.59)
abscessus	0	1	0.00	
abscessus	5	1	365.00	
abscessus	6	4	109.50	
massiliense	11	6	133.83	133.83 (0.00)
**8–30 days**				
abscessus	6	8	54.75	54.75
massiliense	6	15	29.20	30.13 (7.52)
massiliense	4	8	36.50	
massiliense	9	24	27.38	
massiliense	12	23	38.09	
massiliense	8	30	19.47	
**31–180 days**				
abscessus	5	146	2.50	6.18 (3.98)
abscessus	3	42	5.21	
abscessus	6	85	5.15	
abscessus	6	37	11.84	
massiliense	8	42	13.90	7.64 (4.16)
massiliense	9	70	9.39	
massiliense	8	79	7.39	
massiliense	6	171	2.56	
massiliense	11	174	4.61	
massiliense	8	177	3.30	
massiliense	14	74	13.81	
massiliense	14	94	10.87	
massiliense	8	109	5.36	
massiliense	8	112	5.21	

Supplementary Table S2: Estimation of *Mycobacterium abscessus* mutation rates at different time intervals relative to the initial isolate. (Cont.)

Subsp.	No. of SNPs	Interval time (days)	Mutation rate ($\times 10^{-6}$)	Mutation rate ($\times 10^{-6}$), Mean (SD)
**> 180 days**				
abscessus	6	223	1.96	0.82 (0.83)
abscessus	2	191	0.76	
abscessus	5	659	0.55	
abscessus	0	427	0.00	
massiliense	8	216	2.70	2.89 (1.02)
massiliense	12	239	3.67	
massiliense	11	186	4.32	
massiliense	8	336	1.74	
massiliense	12	280	3.13	
massiliense	10	406	1.80	

Note: mutation rate was calculated based on SNP/genome size (5Mb)/year

Confirmatory analysis in Patient 1 with 8 serially isolated strains

No. of SNPs	Interval time (days)	Mutation rate ($\times 10^{-6}$)
6	15	29.20
8	42	13.90
9	70	9.39
8	79	7.39
6	171	2.56
11	174	4.61
8	177	3.30
8	216	2.70

Supplementary Table S3. Frequency and MIC-distribution of isolates with drug resistance-conferring mutations.

**Drug**	**Mutations**	**MIC (µg/mL) distribution for relevant isolate**										**No. of isolates**
**Clarithomycin**		**≤0.06**	**0.12**	**0.25**	**0.5**	**1**	**2**	**4**	**8**	**16**	**>16**	
(CC = 0.25 µg/ml)	No known CLA mutations	1	2	5	6	6	3	3		2		28
	erm(41) T28								3	19	10	32
	rrl A2058G										6	6
	rrl A2059C									2		2
	rrl A2059G										1	1
**Amikacin**		**≤1**	**2**	**4**	**8**	**16**	**32**	**64**	**>64**			
	No known AMK mutations		2	18	32	9	5	3				69
**Ciprofloxacin**		**≤0.12**	**0.25**	**0.5**	**1**	**2**	**4**	**>4**				
(CC = 4 µg/ml)	No known CIP mutations						28	3				31
	gyrB C-41G						8	4				12
	gyrB A-78G						20	6				26
**Linezolid**		**≤1**	**2**	**4**	**8**	**16**	**32**	**>32**				
(CC = 2 µg/ml)	No known LZD mutations			3	3	7	18					31
	rrl C742T						3					3
	rrl A1717G			3	3	6	16					28
	rrl C742T & *rrl* A1717G		2	2								4
	rrl C3042T				1	1	1					3

Supplementary Table S4 Literature of Mutation associated *M. abscessus* drug resistance

Antibiotics	Genes	*E.coli* numbering	*M.abscessus* numbering	Remark	Ref
Amikacin	*rrs*		A-33G		[1]
		T1406A	T1372A		[2]
		A1408G	A1374G		[1,2]
		C1409T	C1375T		[2]
		G1491T	G1455T		[2]
		C1496T	C1460T		[3]
		T1498A	T1462A		[3]
Clarithromycin	*erm*(41)		A-31T		[1]
			C28T		[4-6]
	rrl		G795A		[1]
			T371C		[1]
			T1401C		[1]
			A1932G		[1]
			A2039C		[1]
		A2057G	A2269G		[1]
		A2058G	A2270G		[7,8]
		A2058C	A2270C		[7,8]
		A2058T	A2270T		[7,8]
		A2059T	A2271T		[7,8]
		G2067A	G2279A		[1]

**Antibiotics**	**Genes**	***E.coli* numbering**	***M.abscessus* numbering**	**Remark**	**Ref**
Clarithromycin	*rrl*	A2069A	G2281A		[1]
		A2082C	A2293C		[1]
Ciprofloxacin	*gyrA*	C275T	Ala92Val		[9]
		G286A	Asp96Asn		[9]
		T2287C	Ser763Pro		[1]
Ciprofloxacin	*gyrB*		C-41G		[1]
			A-78G		[1]
			C1474T	Arg492Cys	[9]
			C1727G	Thr576Ser	[1]
Linezolid	*rplC* (L3)		T54A		[10]
Linezolid	*rplD* (L4)		G142A		[10]
			A175P		[10]
Linezolid	*rrl*		G15A	Only R strain	[10]
	(23S rRNA)		T328C	Only R strain	[10]
			G348A	Only R strain	[10]
			C1445T	Only R strain	[10]
			C1582A	Only R strain	[10]
			T2138C	Only R strain	[10]
			A2271C	Only R strain	[10]
			C2432T	Only R strain	[10]
			G3048A	Only R strain	[10]

Antibiotics	Genes	*E.coli* numbering	*M.abscessus* numbering	Remark	Ref
Linezolid	*rrl*		T54A	R+S strain	[10]
	(23S rRNA)		C109T	R+S strain	[10]
			G399A	R+S strain	[10]
			A437G	R+S strain	[10]
			C633T	R+S strain	[10]
			C742T	R+S strain	[10]
			A1717G	R+S strain	[10]
			A2270C	R+S strain	[10]
			T3001C	R+S strain	[10]
			C3042T	R+S strain	[10]

References

1. Lipworth, S.; Hough, N.; Leach, L.; Morgan, M.; Jeffery, K.; Andersson, M.; Robinson, E.; Smith, E.G.; Crook, D.; Peto, T.; et al. Whole-Genome Sequencing for Predicting Clarithromycin Resistance in *Mycobacterium abscessus*. *Antimicrobial agents and chemotherapy* **2019**, *63(1),* e01204-18, doi:10.1128/aac.01204-18.
2. Nessar, R.; Reyrat, J.M.; Murray, A.; Gicquel, B. Genetic analysis of new 16S rRNA mutations conferring aminoglycoside resistance in *Mycobacterium abscessus*. *The Journal of antimicrobial chemotherapy* **2011**, *66*, 1719-1724, doi:10.1093/jac/dkr209.
3. Kim, S.-Y.; Kim, D.H.; Moon, S.M.; Song, J.Y.; Huh, H.J.; Lee, N.Y.; Shin, S.J.; Koh, W.-J.; Jhun, B.W. Association between 16S rRNA gene mutations and susceptibility to amikacin in *Mycobacterium avium* Complex and *Mycobacterium abscessus* clinical isolates. *Sci Rep* **2021**, *11*, 6108-6108, doi:10.1038/s41598-021-85721-5.

4. Kim, S.Y.; Kim, C.K.; Bae, I.K.; Jeong, S.H.; Yim, J.J.; Jung, J.Y.; Park, M.S.; Kim, Y.S.; Kim, S.K.; Chang, J.; et al. The drug susceptibility profile and inducible resistance to macrolides of *Mycobacterium abscessus* and *Mycobacterium massiliense* in Korea. *Diagnostic microbiology and infectious disease* **2015**, *81*, 107-111, doi:10.1016/j.diagmicrobio.2014.10.007.
5. Rubio, M.; March, F.; Garrigó, M.; Moreno, C.; Español, M.; Coll, P. Inducible and Acquired Clarithromycin Resistance in the *Mycobacterium abscessus* Complex. *PloS one* **2015**, *10*, e0140166, doi:10.1371/journal.pone.0140166.
6. Nash, K.A.; Brown-Elliott, B.A.; Wallace, R.J., Jr. A novel gene, erm(41), confers inducible macrolide resistance to clinical isolates of *Mycobacterium abscessus* but is absent from Mycobacterium chelonae. *Antimicrobial agents and chemotherapy* **2009**, *53*, 1367-1376, doi:10.1128/aac.01275-08.
7. Wallace, R.J., Jr.; Meier, A.; Brown, B.A.; Zhang, Y.; Sander, P.; Onyi, G.O.; Böttger, E.C. Genetic basis for clarithromycin resistance among isolates of *Mycobacterium chelonae* and *Mycobacterium abscessu*s. *Antimicrobial agents and chemotherapy* **1996**, *40*, 1676-1681, doi:10.1128/aac.40.7.1676.
8. Pfister, P.; Jenni, S.; Poehlsgaard, J.; Thomas, A.; Douthwaite, S.; Ban, N.; Böttger, E.C. The structural basis of macrolide-ribosome binding assessed using mutagenesis of 23S rRNA positions 2058 and 2059. *Journal of molecular biology* **2004**, *342*, 1569-1581, doi:10.1016/j.jmb.2004.07.095.
9. Lee, S.H.; Yoo, H.K.; Kim, S.H.; Koh, W.-J.; Kim, C.K.; Park, Y.K.; Kim, H.J. The drug resistance profile of *Mycobacterium abscessus* group strains from Korea. *Ann Lab Med* **2014**, *34*, 31-37, doi:10.3343/alm.2014.34.1.31.
10. Kim, S.Y.; Jhun, B.W.; Moon, S.M.; Jeon, K.; Kwon, O.J.; Huh, H.J.; Lee, N.Y.; Shin, S.J.; Daley, C.L.; Koh, W.J. Genetic mutations in linezolid-resistant *Mycobacterium avium* complex and *Mycobacterium abscessus* clinical isolates. *Diagnostic microbiology and infectious disease* **2019**, *94*, 38-40, doi:10.1016/j.diagmicrobio.2018.10.022.
